# Efficacy of Antimicrobial Peptide DP7, Designed by Machine-Learning Method, Against Methicillin-Resistant *Staphylococcus aureus*

**DOI:** 10.3389/fmicb.2019.01175

**Published:** 2019-05-28

**Authors:** Rui Zhang, Zhenling Wang, Yaomei Tian, Qi Yin, Xingjun Cheng, Mao Lian, Bailing Zhou, Xueyan Zhang, Li Yang

**Affiliations:** State Key Laboratory of Biotherapy/Collaborative Innovation Center for Biotherapy, West China Hospital, West China Medical School, Sichuan University, Chengdu, China

**Keywords:** antimicrobial peptides DP7, MRSA, antimicrobial efficacy, blood stream infection, drug development

## Abstract

Antimicrobial peptides (AMPs) provide a promising strategy against infections involving multidrug-resistant pathogens. In previous studies, we designed a short 12 amino acid AMP DP7, using a machine-learning method based on an amino acid activity contribution matrix. DP7 shows broad-spectrum antimicrobial activities both *in vitro* and *in vivo*. Here, we aim to investigate the efficacy of DP7 against multidrug resistant *Staphylococcus aureus* (*S. aureus*) and reveal the potential mechanisms. First, by measuring the killing kinetics of DP7 against *S. aureus* and comparing these results with antibiotics with different antimicrobial mechanisms, we hypothesize that DP7, in addition to its known ability to induce cell wall cation damage, can also exert a full killing effect. With FITC-conjugated or biotin-labeled DP7, we tracked DP7’s attachment, membrane permeation and subsequent intracellular distribution in *S. aureus*. These results indicated that the possible targets of DP7 were within the bacterial cells. Transcriptome sequencing of *S. aureus* exposed to DP7 identified 333 differentially expressed genes (DEGs) influenced by DP7, involving nucleic acid metabolism, amino acid biosynthesis, cell wall destruction and pathogenesis, respectively, indicating the comprehensive killing efficacy of DP7. In addition, the genome sequencing results of the induced DP7 resistant strain *S. aureus* DP7-R revealed two-point mutations in the *mprF* and *guaA* gene. Moreover, in a murine model for MRSA blood stream infection, intravenously treating mice with DP7 showed a good protective effect on mice. In conclusion, DP7 is an effective bactericide for *S. aureus*, which deserves further study for clinical application and drug development.

## Introduction

The increasing frequency of multidrug-resistant bacterial infections is a rising threat to public health. Therefore, there is an urgent need to develop novel antimicrobial agents against infections induced by multidrug-resistant bacteria. Owing to the broad spectrum of antibacterial activity properties, multiple targets and hardly leading to induce bacterial resistance, AMPs are currently under evaluation as a potential defense against multidrug-resistant (MDR) pathogens (Fjell et al., 2012; Deslouches et al., 2013). AMPs are a diverse class of relatively short (12–100 amino acids) cationic peptides (with a net charge of +2 to +9) (Fjell et al., 2012). AMPs have been isolated from both single-cell and multi-cellular organisms; furthermore, it appears that AMPs are produced by all domains of life, acting as an ancient and non-specific innate immune system in defending the host against invading pathogenic organisms, including gram-negative and gram-positive bacteria, fungi, viruses and parasites (Brogden, 2005; Fjell et al., 2012). AMPs have been successfully deployed against bacterial infections in clinical and agricultural settings. Thus, AMPs could be effective antibacterial agents against MDR bacteria without inducing further spread of antimicrobial resistance, which is a common outcome of the use of traditional antibiotics.

Here, the antimicrobial peptide (AMP) DP7, a short 12 amino acid AMP with broad-spectrum antibacterial activities, was chosen to analyze its potential mechanisms for killing *S. aureus*. Compared with other AMPs against *S. aureus* ATCC 25923, such as LL-37 (37 amino acids, MIC = 14 mg/L), pexiganan (22 amino acids, MIC = 32 mg/L) and indolicidin (22 amino acids, MIC = 16 mg/L), DP7 (12 amino acids, MIC = 16 mg/L) was composed of less amino acids with similar or superior minimum inhibitory concentrations (MIC) (Table 1) (Falla et al., 1996; Subbalakshmi and Sitaram, 1998; Dorschner et al., 2001; Zhang et al., 2010; Shen et al., 2012). Compared to AMPs with similar amino acid composition, such as HH2 (12 amino acids, MIC = 64 mg/L), Bac2A (12 amino acids, MIC = 64 mg/L) and Hlf1-11 (11 amino acids, MIC = 64 mg/L), DP7 showed lower MIC values than *S. aureus* (Table 1) (Costa et al., 2014; Yao et al., 2016). Therefore, considering the synthesis cost and antimicrobial potency, DP7 was a potent drug against *S. aureus* in future clinical applications.

**Table 1:** MIC of various AMPs against *S. aureus.*

Peptide	Sequence	Number of amino acids	MIC for ATCC 25923 (mg/L)	MIC for ATCC 33591 (mg/L)	net + n at neutral pH	References
DP7	VQWRIRVAVIRK	12	16^a^	16^a^	+4	
LL-37	LLGDFFRKSKEKIGKEFKRI VQRIKDFLRNLVPRTES	37	14^b^	28^b^	+6	Shen et al., 2012
Pexiganan	GIGKFLKKAKKFGKAFVKILKK	22	32^b^	16^a^	+9	Zhang et al., 2010
HH2	VQLRIRVAVIRA	12	64^b^	64^a^	+3	Wu et al., 2014
Hlf1-11	GRRRRSVQWCA	11	64^a^	64^b^	+3.95	Costa et al., 2014
Bac2A	RLARIVVIRVAR	12	64^b^	43.7^a^	+4	Wu et al., 2014
Indolicidin	ILPWKWPWWPWRR	13	16^b^	64^a^	+3	Wu et al., 2014

*^a^MICs measured in our experiments. ^b^MICs obtained from the references.*

In this study, we aimed to explore the potential mechanism of DP7 killing *S. aureus.* First, we compared the killing curves of DP7 with other antibiotics whose mechanisms are well-studied, confirming that DP7 presented killing effects. Next, we analyzed the effects of DP7 on nucleic acid metabolism, amino acid biosynthesis, cell wall and pathogenesis of *S. aureus* at the transcriptome level. Genome sequencing of the DP7-resistant *S. aureus* strain DP7-R showed two-point mutations in the *mprF* and *guaA* genes. Furthermore, DP7 showed a reduced inhibitory effect on *S. aureus* RN4220 (a natural *sigB* deletion strain) compared with *S. aureus* ATCC 25923. As we expected, DP7 showed favorable therapeutic efficacy in a lethal systemic MRSA infectious mouse model.

## Materials and Methods

### Bacterial Strains

Three *S. aureus* strains, the methicillin-sensitive strain ATCC 25923 (MSSA), the methicillin-resistant strain ATCC 33591 (MRSA), the *sigB*-defective strain RN4220 and *Escherichia coli* (ATCC 25922), as well as the *Pseudomonas aeruginosa* (PAO1) strain were all obtained from the American Type Culture Collection (ATCC, Rockville, MD, United States), and the clinical *S. aureus* isolates were obtained from Southwest Hospital of China (Sichuan, China). This experimental program was approved by the “West China Hospital Review Committee” and written informed consent was obtained from the patients, who volunteered.

### Peptides and Reagents

DP7 (VQWRIRVAVIRK), DP8 (VQLRIRVCVIRK), DP10 (VQLRIRVCVIRK), HH2 (VQLRIRVCVIRK), pexiganan (GIGKFLKKAKKFGKAFVKILKK), FITC-labeled DP7 and biotin-labeled DP7 were synthesized by Shanghai Science Peptide Biological Technology (Shanghai, China) using fluorenylmethyloxycarbonyl chemistry. The synthesized peptides were purified by high performance liquid chromatography (HPLC) to 99% purity, and mass spectrometry was used to confirm their molecular weights. Vancomycin, gentamicin, noroxin, rifampicin, ampicillin and erythromycin were obtained from Dalian Medium Biological Technology Co. Ltd. (Dalian, China).

### Susceptibility Testing and *in vitro* Time-Killing Assays

The susceptibility testing methods were performed as previously described (Wiegand et al., 2008). Briefly, the logarithmic growth-phase bacteria (ATCC 25923 and ATCC 33591) were treated with different drug concentrations at 37°C for 22 h, and the OD_600_ values were subsequently measured to determine the minimum inhibitory concentration (MIC). After MIC determination, the culture medium in all wells where no visible bacterial growth was observed was inoculated in a nutrient agar plate and cultured overnight at 37°C. The minimum bactericidal concentration (MBC) endpoint is the lowest concentration of antibacterial agent that kills > 99.9% of the initial bacterial population where no visible growth of bacteria was observed on nutrient agar plates. Peptides and antibiotics at concentrations corresponding to 1, 2, and 4× MIC (as determined above) were added to bacterial samples (10^6^ CFU/mL) during early log phase to mid-log phase growth. Subsequently, the bacterial samples were counted at different time points (0, 1, 2, 4, 6, 8, 18, and 24 h).

### Confocal Microscopy Assay

Exponential-phase bacteria (10^8^ CFU/mL) were incubated with 1× MIC FITC-DP7 for 30 or 60 min at 37°C. Next, the unbound peptides were removed by extensive washing in PBS (three times) on a shaker. The imaging was carried out using a Zeiss LSM 800 confocal laser scanning microscope (CLSM, Carl Zeiss, Oberkochen, Germany) with a 63× oil objective. Bacteria treated with PBS was served as a control in this experiment.

### Immunoelectron Microscopy

Mid-log phase bacterial cultures of *S. aureus* ATCC 25923 (1 × 10^8^ CFU/mL) were treated with biotin-DP7 at 37°C in 10 mM sodium phosphate buffer (pH = 7.4, 5 mM of glucose, 150 mM of NaCl). After incubation for 1 h, the samples were washed three times with phosphate buffer, and then the *S. aureus* were fixed with 2.5% glutaraldehyde at 4°C. The *S. aureus* were then centrifuged and resuspended in water mixed with 2% agar. Small blocks of agar (approximately 2 Cubic millimeter) (Sigma-Aldrich) containing the samples were dehydrated by soaking in increasing concentrations of ethanol, infiltrated with LR White M acrylic resin (Sigma-Aldrich), and embedded in alginate capsules following procedures previously published (Podda et al., 2006). Ultra-thin sections (100 nm) were cut from the embedded samples, mounted on 300 mesh copper grids and incubated at room temperature for 1 h with drops of 0.1 M of Tris-buffered saline solution (TBS) with pH 7.4, containing 1% (w/v) BSA. After washing three times in PBS, the sections were incubated with a 1:50 dilution of gold-conjugated streptavidin (10 nm gold) (BoHua company, Shanghai, China) for 1 h, and washed three times with PBS to remove the free streptavidin. Non-DP7-treated cells acted as a control in this experiment. Finally, the samples were analyzed by transmission electron microscope (TEM) (Tecnai G2 F20 S-Twin; FEI).

### RNA-seq Library Preparation and Analysis

The quality of the RNA was verified using a Bioanalyzer (Agilent Technologies, Santa Clara, CA, United States) and a NanoDrop spectrometer (Thermo, United States). The A_260/280_ absorbance ration was in the range of 2.0–2.2 and the A_260/230_ ratio was above 1.8. The RNA was sent to Shanghai Personal Biotechnology Co., Ltd. (Shanghai, China) for sequencing using an Illumina Nextseq500 system. We performed quality control analysis by FastQC^1^, and the fastx-toolkit was used to remove potential adapter sequences^2^. The RPKM (reads per kilobase per million reads) values were used to measure the expression level of each gene in the sample. Next, we used Rockhopper to compare the gene values and to analyze the differentially regulated genes. We screened the differentially expressed genes (DEGs) according to the differences in expression and the *p*-values. Finally, the gene functions in the eggNOG genome database (Evolutionary genealogy of genes: Non-supervised Orthologous Groups)^3^,^4^ were used to classify and analyze the gene functional distribution on the macroscopic level.

### Gene Expression Pathway Analysis

Differentially expressed genes were identified by transcriptome sequencing, and genes with *p* < 0.05 were defined as displaying a significant expression difference. After enrichment analysis of the GO (Gene Ontology) and KEGG (Kyoto Encyclopedia of Genes and Genomes) pathways, the differential enrichment *p*-values were corrected by the Bonferroni method (Silva-Vargas et al., 2016). Corrected *p*-values less than 0.05 [-Ln (*p*-value) ≈3] indicated that a pathway was enriched in the DEGs.

### Cell Treatment and RNA Extraction

Mid-log phage *S. aureus* ATCC 25923 (approximately OD_600_ = 0.4–0.6) cells were treated with 16 mg/L DP7 for 1 h and washed three times. The cells were then subjected to the saturated phenol-chloroform extraction method to obtain the total RNA. Untreated cells served as the control. Three independent repeats were performed for this experiment.

### Quantitative Real-Time RT-PCR

According to a standard protocol, total RNA was reverse transcribed using 5 × All-In-One RT MasterMix (Abm, Nanjing, China). The experiment was performed using EvaGreen 2 × qPCR Master Mix (Takara, Dalian, China) and a Lightcycle 96 (Roche) with normal cycling parameters. The cycle threshold values were determined and the relative fold differences were calculated by the 2^-ΔΔCT^ method, using *gyrB* as the reference gene. Three independent repeats were run in triplicate for every experiment.

### Resistance Selection Experiment

According to a published protocol, we chose 4 replicate populations from *S. aureus* ATCC 25923, *S. aureus* ATCC 33591, and cultured them with supplementation with increasing concentrations of DP7 (starting with 1/8× MIC, 2 mg/L), and four control populations were propagated in the medium that lacked any DP7 (Habets and Brockhurst, 2012). For every 48 h, we transferred 2% of each culture to new flasks containing fresh medium with a twofold higher DP7 concentration. When growth was observed at the higher concentration, we used this culture for subsequent inoculation. Aliquots from each population were stored in 20% glycerol at -80°C. Every generation with a higher MIC was tested.

### Whole Genome Sequencing

We extracted the genomic DNA of DP7-resistant bacteria and DP7-sensitive bacteria using the TIANamp Bacteria DNA Kit (QIAGEN) and conducted whole genome sequencing in Novogene (Beijing, China). The original image data files obtained from the high-throughput sequencing (Illumina HiSeq TM2000 and MiSeq sequencing platform) were transformed into the original sequencing sequences (Sequenced Reads) by CASAVA base-pair recognition (Base Calling) analysis. The sequencing data were filtered to remove the sequences containing adapter and low-quality data. The obtained clean data were used for subsequent analysis and the SNPs were detected using SAMTOOLS. Small insertions and deletions (InDels) with lengths less than 50 bp were analyzed. The SNPs/InDels variations in genomic functional regions were analyzed.

### Experimental Murine Model of Blood Stream Infection

A well-characterized murine model of blood stream infection was employed to study the pharmacodynamics profiles of DP7 and compare them with those of vancomycin. Briefly, C57 female mice (body weight 17 g ± 1 g), purchased from Hua Fu Kang Biological Technology Co., Ltd (Beijing, China), were intravenously infected with 1 × 10^8^ CFU/100 μL of MRSA strain ATCC 33591 in the logarithmic growth phase (recovery on defibrinated sheep blood agar, Oxoid, United Kingdom). One hour after infection, the mice were treated with various doses of DP7 (0.5, 1, and 2 mg/kg), while the mice in the control group were injected with the same volume of saline. Vancomycin at 10 mg/kg was used as the positive control. Survival and weight changes were observed every 24 h for 1 week. At 24 and 72 h, organs, including the heart, liver, spleen, lung, kidney, and blood were collected and the bacterial load was counted for each murine. At 72 h, the liver, spleen, lungs, and kidneys were fixed with 4% paraformaldehyde for 1 week, sectioned at 4 μm after processing for paraffin embedding, and then stained with hematoxylin and eosin (H&E). All animal procedures were approved and controlled by the Institutional Animal Care and Treatment Committee of Sichuan University and conducted according to the Animal Care and Use Guidelines of Sichuan University.

### Statistical Analysis

All data comparisons were analyzed using Prism 6.0 with Student’s *t*-test. *p*-values ≤ 0.05 were considered statistically significant, and *p*-values ≤ 0.01 were considered markedly statistically significant.

## Results

### DP7 Showed an Efficient Bactericidal Activity

**FIGURE 1:**
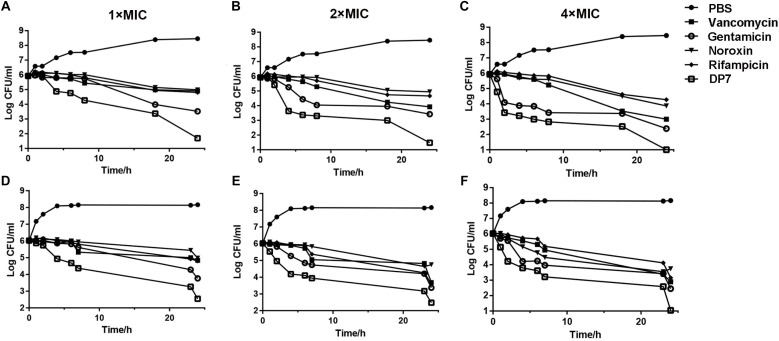
*In vitro* time-killing curves of DP7 compared with some other general antibiotics. Time-killing kinetics of DP7 (open squares) (MIC use here is 16 mg/L), vancomycin (squares) (MIC use here is 0.25 mg/L), gentamicin (open circles) (MIC use here is 0.125 mg/L), noroxin (inverted triangles) (MIC use here is 1 mg/L), and rifampicin (diamonds) (MIC use here is 0.125 mg/L) with untreated bacteria as a growth control (closed circles) at **(A)** 1× MIC, **(B)** 2× MIC, and **(C)** 4× MIC against *S. aureus* ATCC 25923 at 0, 1, 2, 4, 6, 8, 18, and 24 h. (*n* = 3); at **(D)** 1× MIC, **(E)** 2× MIC, and **(F)** 4× MIC against *S. aureus* ATCC 33591 at 0, 1, 2, 4, 6, 8, 18, and 24 h (*n* = 3).

DP7 exhibited broad-spectrum antimicrobial properties (Table 2). We compared DP7’s killing kinetics with some clearly targeted antibiotics. Exposing bacteria to DP7 decreased the CFU/ml (DP7 treatment at 2× and 4× MIC), and the number of living cells decreased 3 logs within 24 h; furthermore, the number of bacteria did not increase over 2 h, indicating that DP7 was a bactericide. The trend in the DP7 killing curve was similar to that resulting from gentamicin exposure (Figure 1). Similarly, the killing curves resulting from exposure to 2× and 4× DP7 MIC showed significantly faster rates of bactericidal activity compared to the rates in bacteria treated with all of the other drugs at higher concentrations. At 4× MIC, DP7 was found to completely stop cell growth within 24 h. As a result, the features of DP7’s killing kinetics suggest a possible comprehensive bactericidal mechanism.

**Table 2:** Antimicrobial assay of DP7.

Strain	MIC (mg/L)	MBC (mg/L)
*S. aureus* (ATCC 25923)	16	64
*S. aureus* (ATCC 33591)	16	64
*E. coli* (ATCC 25922)	16	64
*P. aeruginosa* (PAO1)	16	64
*S. aureus* clinical isolates (MRSA) (SCQ1, SCQ3, S150320016, S3250016, S3396, S3750, S260029) *S. aureus* clinical isolates (MRSA) (S3768)	16 32	128 256

### Intracellular Localization of DP7 in *S. aureus*

**FIGURE 2:**
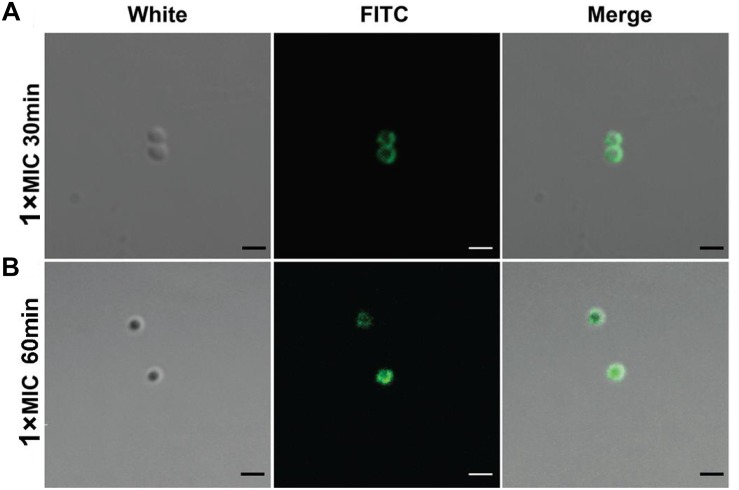
Confocal microscopic imaging shows DP7 aggregation on the cell surface and entry into cells. Confocal microscopic images of *S. aureus* ATCC 25923 after treatment with FITC-DP7 at 1× MIC for **(A)** 30 and **(B)** 60 min. The increasing fluorescence intensity from 30 to 60 min after treatment with the DP7 peptide may indicate progressive membrane damage and pore formation in *S. aureus* ATCC 25923, allowing increased uptake of the fluorescent probe. The scale bar in this figure is 1 μm.

**FIGURE 3:**
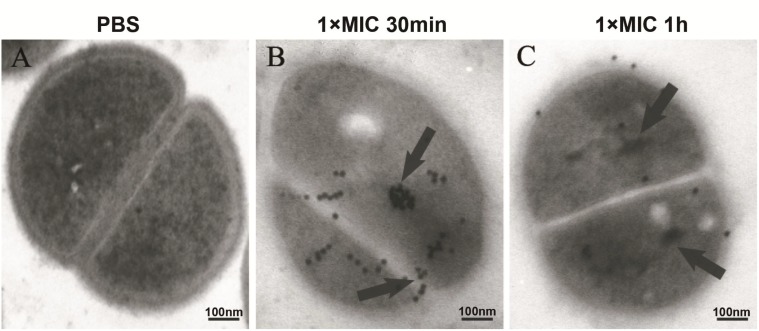
Localization of DP7 in *S. aureus* by transmission electron microscopy. *S. aureus* ATCC 25923 (approximately 1 × 10^8^ CFU/mL) were incubated with PBS **(A)**, or biotin-DP7 (1× MIC) for 30 min **(B)** or for 1 h **(C)**. Fixed bacteria were then incubated with streptavidin (10 nm gold) for 1 h, and then observed under TEM. The length of the bars corresponds to 100 nm. (*n* ≥ 3).

The intracellular localization of DP7 in *S. aureus* cells was examined with confocal laser scanning microscopy (CLSM) and FITC-labeled DP7 (MIC = 32 mg/L). *S. aureus* ATCC 25923 (1 × 10^7^ CFU/mL) was incubated with 1× MIC of FITC-DP7 for 30 and 60 min. Any DP7 accumulation on or within the bacterial cells would result in a green fluorescent signal generation by the FITC that would be visible under the confocal microscope. As shown in Figure 2A, 30 min exposure to FITC-labeled DP7 resulted in an ambient green signal accumulation around the periphery of the cells, suggesting peripheral aggregation of DP7 on the surface of the bacteria (Figure 2A). When the bacteria were exposed to DP7 for 60 min (Figure 2B), the entire cell was encapsulated in green fluorescence, perhaps indicating that DP7 entered the cells (cells treated with PBS or FITC without conjugated DP7 were used as a negative control for this experiment). To further explore this possibility, transmission electron microscopy (TEM) was used. Based on the knowledge that gold-labeled streptavidin binds specifically to biotin, we assumed that the positions of the gold particles might indicate the location of the biotin-conjugated DP7 within the bacteria. Importantly, the control cells treated with PBS and incubated with streptavidin-gold particles showed no gold particles in the view (Figure 3A). However, after culturing bacteria with DP7 (1× MIC) for 30 min, the bacteria showed the accumulation of many bound gold particles (black dots) located in the bacteria, which suggested possible surface adhesion (Figure 3B). After treatment for 1 h, the original sharp black dots became fuzzy, suggesting DP7 penetrated into the cytoplasm. At this time, the cell wall was disrupted, and the inner membranes were obvious. After prolonged peptide incubation, the cell wall became obviously diffuse, thinner and the inner membrane became blurrier than that in the cells without peptide treatment. In the images of cells treated with DP7 for 1 h, there were black dots located in the membranes that looked as if they had “passed through” the cell wall and entered the cytoplasm, and this intracellular accumulation appeared to have a symmetrical distribution (Figure 3C).

### Transcriptomic Analysis of DP7-Treated *S. aureus* ATCC 25923 and *S. aureus* ATCC 33591

In this study, *S. aureus* lab strains, methicillin-sensitive *S. aureus* (MSSA) ATCC 25923 and methicillin-resistant *S. aureus* (MRSA) ATCC 33591 were used as model species to determine the global gene expression change upon DP7 treatment. For *S. aureus*, there are 333 DEGs (250 upregulated genes) from a total of 2820 genes in DP7-treated *S. aureus* ATCC 25923 and 312 DEGs (147 upregulated genes) from a total of 2730 genes in DP7-treated *S. aureus* ATCC 33591, respectively (Supplementary Tables S1, S2). Gene annotations including statistical analysis of the enrichment pathways of *S. aureus* ATCC 25923 and *S. aureus* ATCC 33591 can be found in Supplementary Tables S3, S4.

**FIGURE 4:**
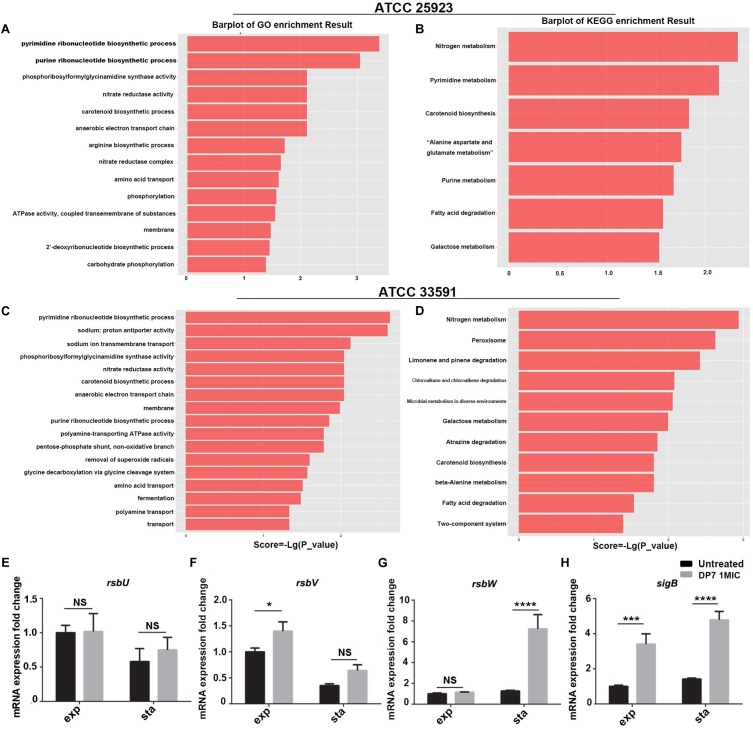
Correlation pathway enrichment of differentially expressed genes after DP7 (1× MIC) treatment of *S. aureus* ATCC 25923 and ATCC 33591. **(A)** GO term enrichment results of DP7-treated ATCC 25923. **(B)** KEGG term enrichment results of DP7-treated ATCC 25923. **(C)** GO term enrichment results of DP7-treated ATCC 33591. **(D)** KEGG term enrichment results of DP7-treated ATCC 33591. **(E–H)** Expression of *sigB*-related genes in *S. aureus* ATCC 25923 (black bars), DP7 (16 mg/L)-treated *S. aureus* ATCC 25923 (gray bars) during exponential (exp) and stationary (sta) phases. Each single row in the heat map corresponds to a single gene. (*n* ≥ 3; ^∗^*p* < 0.05; ^∗∗∗^*p* < 0.001; ^∗∗∗∗^*p* < 0.0001).

First, the impact of DP7 treatment on nucleic acid metabolism were analyzed, and the results showed that DNA and RNA biosynthetic process-associated genes exhibited significant differential expression after DP7 treatment on *S. aureus*, especially for pyrimidine and purine ribonucleotide biosynthetic process-related genes (Figure 4A–D). Notably, genes encoding DNA polymerase subunits, DNA gyrase subunits, DNA topoisomerase subunits and chromosome replication protein DnaD were differentially expressed. Some DNA-directed RNA polymerase submits were found to be differentially expressed after DP7 treatment, including *alpha*-subunit upregulation and *beta*, *delta*, *omega*-subunit downregulation in *S. aureus* ATCC 33591. This is accompanied by upregulation of RNA polymerase sigma factor *sigB* in both two *S. aureus* strains, and RNA polymerase sigma factor *rpoD* in *S. aureus* ATCC 33591. *SigB* was upregulated in DP7-treated cells in both *S. aureus* ATCC 25923 and *S. aureus* ATCC 33591, but there no differential expression was observed with DNA-binding RNA polymerase subunits, which indicated DP7 had no inhibitory activity on RNA polymerase. But RNA methyltransferase was influenced by DP7 in *S. aureus* ATCC 33591. Two of three translation-initiation factor-2 (IF-2) and IF-3 were differentially expressed after DP7 treatment. Downregulation of the A subunits in DNA topoisomerase IV was found in *S. aureus* ATCC 25923 and *S. aureus* ATCC 33591 after DP7-treatment. The expression of topoisomerase I and III were decreased in DP7-treated *S. aureus* ATCC 33591. In *S. aureus* ATCC 33591, DNA gyrase subunit A and IS1272 transposase were downregulated after being treated with DP7. In addition, *carAB* (Carbamoyl-phosphate synthase small chain) in the pyrimidine pathways was downregulated 17- and 10-fold lower than those of the control. Additionally, *pyrC* (Dihydroorotase) was upregulated 18.37-fold higher than that of normal control.

The transcriptomic analysis of *S. aureus* ATCC 25923 and *S. aureus* ATCC 33591 showed that DP7 has effects on amino acid biosynthesis processes in *S. aureus*. From the gene enrichment analyses of DP7-treated *S. aureus*, the precursory pathways responsible for amino acid biosynthesis were noted. These included nitrogen, aromatic compound, amine acid, and carboxylic compound biosynthesis processes. The DEGs encoded for various amino acids were reported, including tryptophan, lysine, valine, leucine, isoleucine, arginine, proline, histidine, alanine, aspartate, glutamate, and tyrosine metabolism. The aromatic family amino acids tryptophan, the lysine in basic amino acids, and aliphatic family amino acids lysine and valine were affected significantly. The expression of DEGs resulted in new metabolisms of stress pathways being activated. Several genes were associated with arginine biosynthetic process pathways, nitrate reductase complex and amino acid transport, lyase, and chorismate’s metabolic process. Similar results were observed in DP7-treated *S. aureus* ATCC 33591. In addition, the generation of peptides/proteins on the downstream processes following influenced amino acids biosynthesis were affected by the treatments as well. Changes of RNA expressions were associated with tRNA ligase activity, 30S and 50S ribosomal proteins, and ribosomal small subunit assembly. The translation-initiation factors (IFs) IF2 and IF3 were downregulated after DP7 treatment in *S. aureus* ATCC 33591.

Next, the effects of DP7 treatment on the cell wall of *S. aureus* and pathogenesis were analyzed. Gene enrichment analyses highlighted that genes encoding for cell membrane and amino acid transport pathways were clearly impacted in DP7-treated *S. aureus*. More than 60 genes were differentially expressed in the two pathways and represented the largest gene sets as compared to other pathways. Moreover, DP7-treated *S. aureus* was reported with changes in the carotenoid biosynthetic process (GO:0016117), phosphoribosylformylglycinamidine syntheses activity (GO:0004642), nitrate reductase activity (GO:0008940), and anaerobic electron transport chain (GO:0019645). In DP7-treated *S. aureus*, two genes (KQ76_RS12545, KQ76_RS02320) associated with cell wall synthesis were downregulated in *S. aureus* ATCC 25923 and *S. aureus* ATCC 33591. In addition, DP7 treatment resulted in upregulation of the carotenoid biosynthetic pathway (GO:0016117)-related genes and lysostaplin resistance protein A (lyra, KQ76_RS13890) and toxin (KQ76_RS11290).

### The Growth Inhibitory Rate of DP7 on *S. aureus* RN4220 Was Lower Than That in *S. aureus* ATCC 25923

**FIGURE 5:**
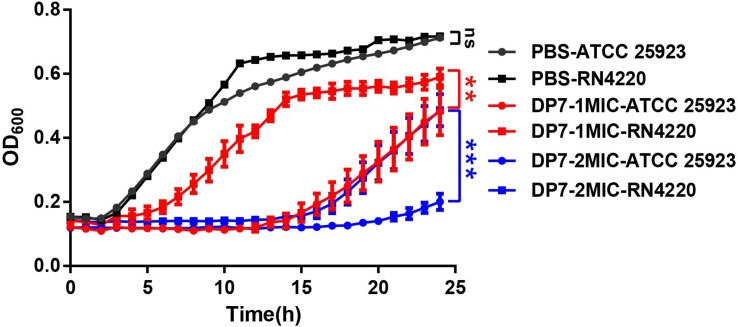
The growth inhibition rates of DP7 in *S. aureus* RN4220 were lower than in *S. aureus* ATCC 25923. Cells were cultured at 37°C with aeration using a Bioscreen C automated growth analysis system (*n* = 3). In this study, the OD values were recorded every 3 min, and the hourly OD values are plotted in this figure. ^∗∗^*p* < 0.01; ^∗∗∗^*p* < 0.001.

In this section, we used RT-PCR to verify the consistency of *sigB*-related gene expression and the results of the transcriptome sequencing in DP7-treated *S. aureus* ATCC 25923 (Table 3) (Figure 4E–H). Primers were listed in Supplementary Table S5. A *sigB*-defective strain was used to further verify our results. *S. aureus* RN4220, with an 11-bp defect in *rsbU*, was described as a natural *sigB*-defective strain (Kullik et al., 1998; Rachid et al., 2000; Giachino et al., 2001). The growth curves of *S. aureus* ATCC 25923 and *S. aureus* RN4220 were tested in the presence or absence of DP7 to clarify whether DP7 killing *S. aureus* was related to the *sigB* synthesis regulon. Cells were grown in triplicate at 37°C with aeration using a Bioscreen C automated growth analysis system. In this experiment, the OD values were measured every 3 min, and only the hourly OD values were used for plotting. As seen in Figure 5, the growth inhibitory rate of DP7 on *S. aureus* RN4220 was lower than that in *S. aureus* ATCC 25923 at 1× MIC and 2× MIC, demonstrating that *sigB* plays an important role in the DP7 killing of *S. aureus*.

**Table 3:** The fold changes determined by RNA-seq of some *sigB*-related genes.

Gene	Description	Fold-
name		-change
**Rank upregulated**		
*sigB*	RNA polymerase sigma factor s*igB*	5.37
*rsbU*	Serine phosphatase	1.20
*rsbV*	*Anti-sigma-B* factor antagonist	2.72
*rsbW*	Serine-protein kinase *rsbW*	3.70
*purR*	*LacI* family transcriptional regulator	1.53
*guaA*	GNAT family acetyltransferase	1.34
*graS*	Sensor histidine kinase *graS*	1.38
*graR*	Response regulator protein *graR*	1.66
*mprF*	Phosphatidylglycerol lysyltransferase	1.72
*agrA*	Histidine kinase	1.81
*agrB*	Accessory gene regulator protein B	1.21
**Rank downregulated**		
*purF*	Amidophosphoribosyltransferase	-3.83
*purN*	Phosphoribosylglycinamide formyltransferase	-2.45
*purQ*	Phosphoribosylformylglycinamidine synthase subunit *purQ*	-4.58
*purL*	Phosphoribosylformylglycinamidine synthase subunit *purl*	-5.23
*purM*	Phosphoribosylformylglycinamidine cyclo-ligase	-3.00
*purK*	N5-carboxyaminoimidazole ribonucleotide synthase	-2.17
*purC*	Phosphoribosylaminoimidazole-succinocarboxamide synthase	-2.72
*guaB*	Inosine-5′-monophosphate dehydrogenase	-1.71
*guaC*	GMP reductase	-2.24
*dnaN*	DNA polymerase III subunit *beta*	-1.05
*rpoA*	DNA-directed RNA polymerase subunit *alpha*	-1.36

### DP7-R (DP7-Resistant *S. aureus* Strain) Showed Point Mutations at *mprF* and *guaA*

**FIGURE 6:**
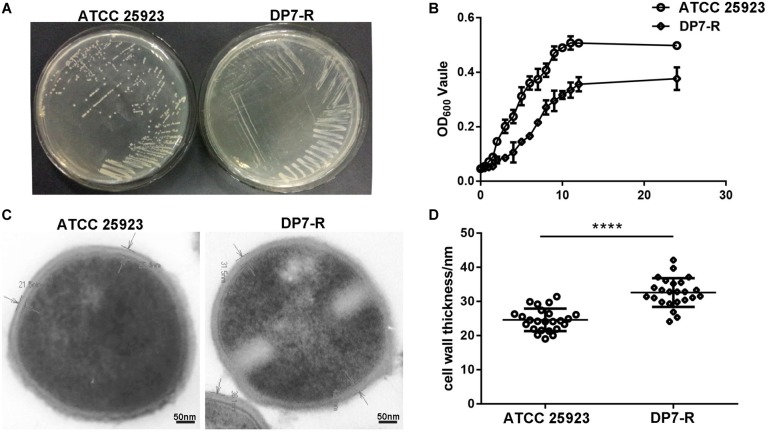
DP7-R showed a slower growth rate and thicker cell wall compared with wild-type DP7-sensitive strains. **(A)** Comparison of wild-type *S. aureus* ATCC 25923 and DP7-R. **(B)** DP7-R shows a slower growth rate than ATCC 25923. **(C)** The cell wall thicknesses of *S. aureus* ATCC 25923 and DP7-R. **(D)** Cell wall thickness statistics in different cells (approximately 20 cells) in several fields of view (*n* ≥ 3; ^∗∗∗∗^*p* < 0.0001).

After the 6 months of resistance selection experiments, the *S. aureus* strain DP7-R (MIC = 64 mg/L) (obtained from ATCC 25923 through DP7-induced drug resistance, while ATCC 33591 did not produce DP7-resistant strains) was selected from this experiment. After serial passage in normal medium, DP7-R was still resistant to DP7. *S. aureus* DP7-R carried two-point mutations in *mprF* (N353K and S567L) and one-point mutation in *guaA* (D450Y) in its genome sequence (Table 4). It was worth noting that *S. aureus* DP7-R had a slower growth rate compared with *S. aureus* ATCC 25923 (Figure 6A,B). Based on the upregulation of cell wall-related genes after DP7 treatment, we characterized the thickness of the cell wall in *S. aureus* ATCC 25923 and DP7-R. Consistent with the described physiology, the cell wall of *S. aureus* DP7-R was thicker than that of *S. aureus* ATCC 25923 (Figure 6C,D). We also tested the MICs of other AMPs against DP7-R, and the results revealed that DP7-R showed resistance to other peptides, such as HH2 (1× MIC to 4× MIC) (MIC = 64 mg/L), Pexiganan (1× MIC to 4× MIC) (MIC = 32 mg/L), DP8 (1× MIC to 4× MIC) (MIC = 16 mg/L), DP10 (1× MIC to 4× MIC) (MIC = 64 mg/L), and Hlf1-11 (1× MIC to 4× MIC) (MIC = 64 mg/L) (Table 5), while DP7-R was not resistant to some antibiotics, such as ofloxacin (1× MIC to 2× MIC) (MIC = 0.25 mg/L), moxifloxacin hydrochloride (1× MIC to 1× MIC) (MIC = 0.125 mg/L), norfloxacin (1× MIC to 2× MIC) (MIC = 1 mg/L), vancomycin (1× MIC to 1× MIC) (MIC = 0.25 mg/L), azithromycin (1× MIC to 1× MIC) (MIC = 0.125 mg/L), rifampin (1× MIC to 1× MIC) (MIC = 0.125 mg/L), but was resistant to gentamicin (1× MIC to 4× MIC) (MIC = 0.0625 mg/L).

**Table 4:** Single nucleotide changes located in open reading frames.

Strain and site^a^	Base change	Amino acid change	Locus	MIC^b^ (mg/L)
DP7-R				
1354274	T→G	N353K	*mprF*	64
1354918	C→T	S567L	*mprF*	
396702	G→T	D450 Y	*guaA*	

*^a^Location of mutations in the S. aureus strain ATCC 25923 sequence. ^b^The MICs with the highest frequencies for each species. Values are averages from three independent experiments performed in triplicate.*

**Table 5:** Determination of MICs of a variety of antimicrobial peptides in ATCC 25923 and DP7-R.

	MIC (mg/L)					
Strains	DP7	HH2	Pexiganan	DP8	DP10	Hlf1-11
ATCC 25923	16	64	32	16	64	64
DP7-R	64	256	128	64	256	256

### DP7 Showed Favorable Therapeutic Efficacy in a Mouse Model of MRSA Blood Stream Infection (BSI)

**FIGURE 7:**
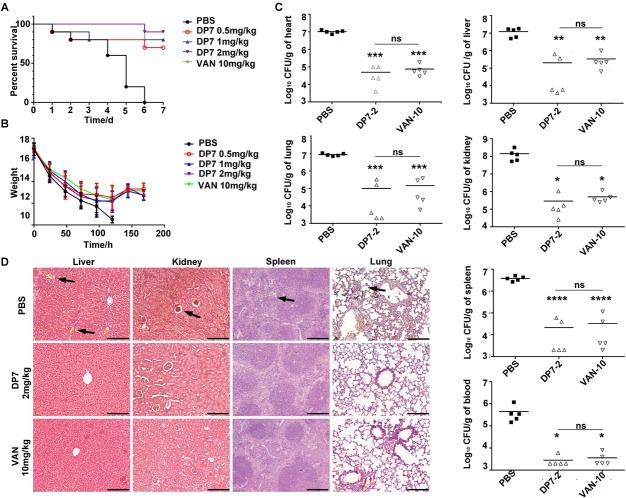
Experimental murine model of blood stream infection. C57 female mice were intravenously injected with 1 × 10^8^ CFU/100 μL ATCC 33591, and antimicrobial drugs were intravenously administered 1 h after injection. The treatments of DP7 doses were 0.5, 1, and 2 mg/kg. PBS and 10 mg/kg of vancomycin acted as negative and positive control, respectively. The bacterial loads at 72 h after infection were counted. Every symbol in Figure 1C represents the viable bacterial counts determined for each mouse, while the average values for each group is represented by the horizontal lines. **(A)** The survival rate and **(B)** weight changes after DP7 treatment (*n* = 10). **(C)** CFU counts/g of organs after treatment with 2 mg/kg DP7 or 10 mg/kg vancomycin at 24 h and 72 h (*n* = 5). **(D)** Histology of major organs of mice following lethal MRSA infection. The length of the bars corresponds to 100 μm.

Blood stream infections (BSIs) have been an important cause of mortality in recent years, especially in *S. aureus* (20%) BSIs (Wisplinghoff et al., 2004). In this study, the therapeutic efficiency of DP7 was tested in a lethal model of MRSA BSI. The inoculums were optimized to produce lethal infection, with death occurring within 1-week post-infection. Typical physical and behavioral presentations associated with severe MRSA infection (such as decreased body weight, increased eye secretions, and decreased activity) were observed, and the infection was confirmed by testing MRSA in organ homogenates. The results showed that DP7 at doses of 0.5, 1, and 2 mg/kg led to 70, 80, and 90% reductions, respectively, in the lethality of the infection up to day 7 post-infection (Figure 7A). The protection level of DP7 at 1 mg/kg was equivalent to 10 mg/kg vancomycin in this infection model (Supplementary Figure S1). In addition, the survival of the systemically infected mice was significantly enhanced after DP7 treatment. Furthermore, DP7-treated infected mice showed slower weight loss than the control and showed recovery of weight 5 days post-infection, as occurs with vancomycin treatment (Figure 7B). The bacterial load in the heart, liver, spleen, lung, kidney, and blood was determined after 72 h of therapy. Compared with PBS-treated mice at the end of the therapy, 2 mg/kg DP7 and 10 mg/kg vancomycin led to statistically significant reductions in the bacterial load (Figure 7C). Organs from the infected control mice showed multiple lesions, damage to tissue structure, infiltration of erythrocytes and heavy alveolar congestion in the lungs (black arrow). This type of histological damage in DP7 (2 mg/kg)-treated mice was significantly minimal compared to the control mice (Figure 7D).

## Discussion

Due to serious drug resistance, conventional antibiotics are becoming increasingly ineffective. Thus, it is imperative to develop new antibacterial strategies. AMPs are used to treat multidrug-resistant bacteria because of their broad-spectrum antimicrobial activities, multi-targets and difficulty in inducing drug resistance. However, the synthetic cost of AMPs may limit their application. Therefore, it is still worth developing a more efficient and cost-effective broad-spectrum amplifier. Here, we are concerned with DP7—compared with other AMPs such as LL-37, pexiganan and indolicidin, DP7 has less amino acid composition and the MIC for *S. aureus* is not higher than these AMPs. Compared with Hlf1-11, Bac2A and HH2, DP7 has the same amino acid composition, and the MIC of DP7 to *S. aureus* is lower or equal to these AMPs. Therefore, considering antimicrobial activity and cost-effectiveness, DP7 deserves further study.

In the present study, we aimed to explore the DP7’s potential mechanisms for killing *S. aureus*. DP7 was designed based on the identified peptide HH2 and optimized with an amino acid-based prediction method by determining the contribution of each amino acid to the antimicrobial activity of the peptide (Wu et al., 2014). Our method, based on a novel peptide prediction method and determination of the quantitative structure-activity, was more accurate at determining the efficacy of the peptide but was not helpful in identifying potential targets (Ostberg and Kaznessis, 2005; Wu et al., 2014). Considering the amino sequence and lengths of DP7, the chances that it has a secondary structure is unlikely, and evidence of self-assembly is absent for DP7. Therefore, it is difficult to explain the uneven attachment and distribution of DP7 on the *S. aureus* cell surface. Furthermore, the similarities in the killing kinetics shared between DP7 and ribosome-targeted or nucleic synthesis inhibitory antibiotics (rather than cell wall-active antibiotics) prompted us to explore if there are additional DP7 targets (Schneider et al., 2010). Then we mapped the physiological changes after DP7 treatment via transcriptomic analysis. Through the whole genome sequencing analysis, we examined the changes in physiological functions in *S. aureus* before and after induced DP7 resistance. One of the goals of the present study was to outline the possible signaling pathways modulated by DP7 which could help to better understand its possible mode of action.

To analyze the global effects of DP7 in *S. aureus*, we performed comparative transcriptome analysis on MSSA strain *S. aureus* ATCC 25923 and the MRSA strain *S. aureus* ATCC 33591 with or without DP7 exposure. Interestingly, DP7 had drastic effects on *S. aureus*’s pyrimidine and purine biosynthesis metabolism. Specifically, *carAB*, a component of the pyrimidine pathway, was downregulated by 17- and 10-fold relative to the control. As a part of the arginine and pyrimidine pathways, the *carAB* gene encodes the enzyme carbamoylphosphate synthetase (CPS), which catalyzes the synthesis of carbamoylphosphate (Piette et al., 1984; Martinussen and Hammer, 1998; Nicoloff et al., 2001; Butcher et al., 2016). Carbamoylphosphate is a common intermediate in arginine and pyrimidine biosynthesis (Werner et al., 1985). Although the exact regulated mode of *carAB* in *S. aureus* is still unclear, similar studies performed on other gram-negative microbes suggest that arginine is likely involved in the regulation (Sekowska et al., 2001). Regulation of the *carAB* operon in *P. aeruginosa* is controlled by arginine and pyrimidines at the transcriptional level, possibly through an attenuation mechanism (Lu et al., 1997; Butcher et al., 2016). Further studies on *E. coli* identified an arginine box located upstream of the control region of *carAB* that served as the initiation site, but which was blocked upon arginine recognition (Piette et al., 1984; Crowell et al., 1987; Alwan et al., 2017). Due to the arginine-rich nature of DP7’s sequence, combined with the results of our earlier study proving that DP7 possesses no secondary structure and has a low chance of self-assembly, we speculated that the arginine box might be recognized by DP7 to inhibit *carAB* transcription. This possibility must be demonstrated through future experiments, as no evidence has suggested the existence of such arginine boxes in *S. aureus* or any other gram-positive species. Interestingly, while almost all the genes involved in the de novo synthesis of pyrimidines were downregulated, the expression of *pyrC* was upregulated by 18.37-fold compared to its level in the normal control. This result indicates that these pathways are tightly regulated and balanced in the cell. Further studies are warranted to investigate the mechanisms of nucleotide regulation in *S. aureus*, e.g., deciphering the role of end-product repression in these pathways.

During the induction of cell envelope stress, it is reasonable to assume that AMPs could bind to molecules located on the cell wall or membrane. Some of these molecules might act as global regulators that sense envelope stress and react as “effectors” after stimulation due to sustained exposure (Hu and Coates, 1999). The stress-activated sigma factor B (*sigB*) was reported to be involved in resistance to cell wall-active antibiotics such as beta-lactamase and vancomycin. There are two primary regulators of *sigB, rsbV* and *rsbW. RsbW* can bind to *sigB* protein, which renders *sigB* unavailable to RNA polymerase, while *rsbV* is the *sigB* release factor. Under stressful conditions, *rsbW* binds to non-phosphorylated *rsbV* and releases *sigB* (Voelker et al., 1996; Thompson et al., 2015). In this study, we observed that DP7 perturbed the *rsbU-sigB* transcription system, which could partly explain the strong effects of DP7 on gene expression, bacteria growth or infectivity. Related to the *sigB* regulon, DP7 increased the *sigB* transcriptional level by 5.37-fold in *S. aureus* ATCC 25923. The increased *sigB* positively regulates the purine operon repressor (*purR*), then inhibits the expression of the downstream pur genes, and downregulates the expression of the *gua* genes, thus affecting the synthesis of GMP. Inhibition of GMP synthesis inhibits the expression of DNA polymerase III subunit beta (*dnaN*) and DNA-directed RNA polymerase subunit alpha (*rpoA*), thereby inhibiting DNA and RNA synthesis (Table 3). Combined with the observation that DP7-R was found to develop a thicker cell wall upon ongoing DP7 exposure, our data were consistent with the fact that artificial overexpression of *sigB* in *S. aureus* resulted in a thickened cell wall (Mishra et al., 2009). Furthermore, other cell wall-targeted antibiotics have also been proven to alter *S. aureus* virulence by affecting *sigB*-regulated biofilm formation. Even though no specific DP7 target was identified in this study, the impaired killing effect of DP7 in the *sigB*-negative *S. aureus* strain RN4220 suggested that sigB might be one of the potential targets of the DP7.

DP7-R carried two-point mutations in *mprF* (N353K and S567L) and single point mutation in *guaA* (D450Y). The upregulation of *mprF* reduced the negative net charge of the cell membrane via the formation of lysyl-phosphatidylglycerol (Lys-PG), which limits the attraction to AMP (Ernst and Peschel, 2011; Rubio et al., 2012). The two point mutations in *mprF* have not been mentioned in previous studies, thus they may indicate a new cause of reduced membrane charge (Quinn et al., 2007). *GuaA*, known as an integrated hot spot for genetic islands, transposons and bacteriophages in bacterial species, has not been reported to be associated with peptide resistance (Mulhbacher et al., 2010; Wipf et al., 2015). We assumed that the point mutant in *guaA* may be related to a new mechanism of peptide resistance, which will be clarified in our future studies. Furthermore, DP7-R showed multi-peptide resistance, which may indicate that the activated *sigB* system may also enhance the tolerance of *S. aureus* to other peptides. In the case of MSI-78, which was previously studied by Habets and Brockhurst (2012), *S. aureus* could develop a stable 10- to 50-fold increase in the MIC. However, in our study, *S. aureus* showed, at most, an 8-fold increase in DP7 resistance, which may indicate that the development of DP7 resistance by bacteria may be difficult.

Proliferation of *S. aureus* in the blood represents one of the most dangerous stages of *S. aureus* infection. Considering the inhibitory effect of DP7, we examine the possible therapeutic efficacy of DP7 in *S. aureus* blood infection. We tested the potency of DP7 in a murine model of BSI by counting the survival of DP7-treated animals. Following intravenous administration, DP7 showed an effective protective effect on mice with blood infection. In summary, by conducting the murine model of BSI, we demonstrate that DP7 could be a promising strategy for fighting *S. aureus* infection in the future. These experiments demonstrated that DP7, a computer-designed AMP, may be effective in a more clinical scenario.

## Ethics Statement

All animal procedures were approved and controlled by the Institutional Animal Care and Treatment Committee of Sichuan University and conducted according to the Animal Care and Use Guidelines of Sichuan University. In addition, we declare all animal experiments comply with the ARRIVE guidelines.

## Author Contributions

RZ and ZW coordinated the whole project and wrote the article. YT and QY synthesized and purified some peptides. BZ, XC, and ML performed the bioinformatics analysis. XZ performed *in vitro* experiments for MIC. The manuscript was written with contributions from all the authors. All authors have given approval to the final version of the manuscript.

## Conflict of Interest Statement

The authors declare that the research was conducted in the absence of any commercial or financial relationships that could be construed as a potential conflict of interest.

## Footnotes

1 http://www.bioinformatics.babraham.ac.uk/projects/fastqc/

2 http://hannonlab.cshl.edu/fastx_toolkit/index.html

3 http://www.ncbi.nlm.nih.gov/COG/

4 http://eggnog.embl.de/version_3.0/
