# Supplementary material for: Efficacy of Antimicrobial Peptide DP7, Designed by Machine-Learning Method, Against Methicillin-Resistant Staphylococcus aureus
Source: Front Microbiol. 2019 May 28;10:1175. doi: 10.3389/fmicb.2019.01175 (PMC6546875; doi:10.3389/fmicb.2019.01175)
Supplement: Supplementary file 5 [file Data_Sheet_1.pdf]

Table s1: Analysis of differentially expressed genes of ATCC 25923 treated with 1MIC DP7 or PBS

Table s2: Analysis of differentially expressed genes of ATCC 33591 treated with 1MIC DP7 or PBS

Table s3: Gene ontology enrichment analysis of differentially expressed genes in ATCC 25923 treated with 1MIC DP7 or PBS

Table s4: Gene ontology enrichment analysis of differentially expressed genes in ATCC 33591 treated with 1MIC DP7 or PBS

Table s5: qRT-PCR primers used in this study

Name	Sequence(5'→3')a	Name	Sequence(5'→3')a
sigB -RT-F	AATGGTCATCTTGTTGTCCCT	*sigB* -RT-R	ACCGCTGAATTAGAGCGTTCA
rsbU-RT-F	AGCCATCACTCGGTAATTGTGA	*rsbU*-RT-R	TTCAGTGGCGGCACAAAAAG
rsbV-RT-F	CCTACGAATAAACCTAAACCTGTCG	*rsbV*-RT-R	ACGAAGTTAAAGTCGGTGGAGA
rsbW-RT-F	TGTTTAACTGCATTTGTCACAGC	*rsbW*-RT-R	TTTATCGAAATGCGCGTGCC
gyrB-RT-F	ATTGCACAGCCACCGTTGTA	*gyrB*-RT-R	CGGTGCTCAGGGTTCATTGT

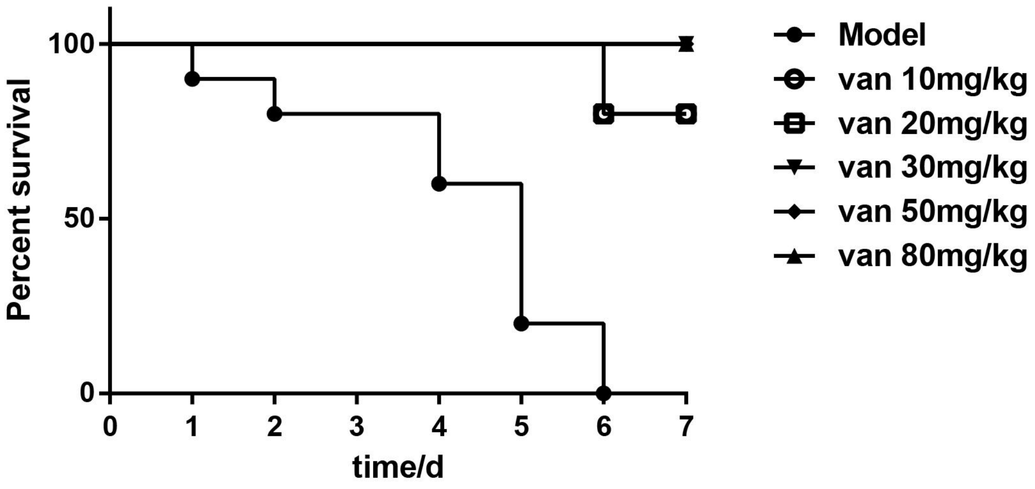

Figure S1. Experimental murine model of blood stream infection. C57 female mice were infected with 1×10^{8} CFU of MRSA ATCC 33591 by intravenous injection, and different concentrations of vancomycin were administered 1 h after injection. Then the survival of mice was counted every 24 hours.
